# Whole genome resequencing of *Botrytis cinerea* isolates identifies high levels of standing diversity

**DOI:** 10.3389/fmicb.2015.00996

**Published:** 2015-09-24

**Authors:** Susanna Atwell, Jason A. Corwin, Nicole E. Soltis, Anushryia Subedy, Katherine J. Denby, Daniel J. Kliebenstein

**Affiliations:** ^1^Department of Plant Sciences, University of California, DavisDavis, CA, USA; ^2^School of Life Sciences and Warwick Systems Biology Centre, University of WarwickCoventry, UK

**Keywords:** *Botrytis cinerea*, genome resequencing, standing genetic variation, diversity, vegetative incompatibility loci

## Abstract

How standing genetic variation within a pathogen contributes to diversity in host/pathogen interactions is poorly understood, partly because most studied pathogens are host-specific, clonally reproducing organisms which complicates genetic analysis. In contrast, *Botrytis cinerea* is a sexually reproducing, true haploid ascomycete that can infect a wide range of diverse plant hosts. While previous work had shown significant genomic variation between two isolates, we proceeded to assess the level and frequency of standing variation in a population of *B. cinerea*. To begin measuring standing genetic variation in *B. cinerea*, we re-sequenced the genomes of 13 different isolates and aligned them to the previously sequenced T4 reference genome. In addition one of these isolates was resequenced from four independently repeated cultures. A high level of genetic diversity was found within the 13 isolates. Within this variation, we could identify clusters of genes with major effect polymorphisms, i.e., polymorphisms that lead to a predicted functional knockout, that surrounded genes involved in controlling vegetative incompatibility. The genotype at these loci was able to partially predict the interaction of these isolates in vegetative fusion assays showing that these loci control vegetative incompatibility. This suggests that the vegetative incompatibility loci within *B. cinerea* are associated with regions of increased genetic diversity. The genome re-sequencing of four clones from the one isolate (Grape) that had been independently propagated over 10 years showed no detectable spontaneous mutation. This suggests that *B. cinerea* does not display an elevated spontaneous mutation rate. Future work will allow us to test if, and how, this diversity may be contributing to the pathogen's broad host range.

## Introduction

Assessing how genomic variation may play a role in assisting pathogens to adapt to the difficulties of their corresponding hosts has largely been driven by cross-species comparisons as illustrated by the efforts on generating 1000 or more genomes for entire Kingdoms (for example 1000 Fungal Genomes Project, 10,000 Microbial Genomes Project, 100 K Pathogen Genome Project). In fungi, published pathogen genomes include numerous potential plant and animal pathogen species such as, *Aspergillus fumigatus* (Nierman et al., 2005), *Cryptococcus neoformans* (Loftus et al., 2005), *Pythium ultimum* (Lévesque et al., 2010), *Sclerotinia sclerotiorum* (Amselem et al., 2011), *Taphrina deformans* (Cissé et al., 2013), and *Botrytis cinerea* (Amselem et al., 2011; Staats and van Kan, 2012; Blanco-Ulate et al., 2013). This cross-species comparative genomics has identified some key components of host/pathogen interactions such as plant pathogens typically having more carbohydrate metabolic genes than animal pathogens (Porcel et al., 2014).

Cross-species comparisons are largely constrained to look at genome changes that are old and likely fixed within the species being analyzed (Hudson et al., 1987). In contrast, host/pathogen interactions are continuously changing and evolving. To identify these younger changes requires whole-genome re-sequencing of numerous individuals within a host or pathogen species to find the standing genetic variation that could be contributing to variation within current host-pathogen interactions. While many re-sequencing projects are under way such as the 20,000 Global pneumococcal project and several *Saccharomyces cerevisiae* re-sequencing projects (Wilkening et al., 2013), currently published sequence is limited to the host of any host/pathogen interaction, i.e., from Humans (Altshuler et al., 2010) and *Arabidopsis* (Long et al., 2013). Available datasets investigating genetic diversity in pathogens have tended to follow gene specific approaches (Baltrus et al., 2011; Guo et al., 2014) rather than unbiased whole genome investigations. Similarly, these approaches have focused solely on nuclear genes with minimal assessment of mitochondrial genome variation. A number of studies are beginning to show that genetic variation within the organellar genomes of diverse species can epistatically interact with the variation in the nuclear genome to control adaptive traits suggesting that it is necessary to consider that organelle variation may alter host-pathogen interactions (Etterson et al., 2007; Tang et al., 2007, 2013; Wolf, 2009; Dowling et al., 2010; Tan et al., 2012; Joseph et al., 2013a,b, 2015). Thus, there is restricted genomic information available to aid in the identification of causal polymorphisms in the pathogens controlling the host-pathogen interaction. This indicates a stark need for studies providing detailed whole genome measurements of genetic diversity in pathogens for both the nuclear and mitochondrial genomes.

*B. cinerea* is a necrotrophic fungal plant pathogen that has been a focus of gene variation studies over the past several decades. It has a genome of approximately 41–42 Mbp spread across 16 chromosomes (Shirane et al., 1989; Amselem et al., 2011; Staats and van Kan, 2012; Blanco-Ulate et al., 2013). This fungus has the capacity to infect and cause disease in living tissue of diverse plants ranging from numerous dicots to gymnosperms and bryophytes (Darvas et al., 1978; Coley-Smith et al., 1980; Lorbeer, 1980; Ponce de León et al., 2007; Williamson et al., 2007; Ponce de León et al., 2012). This host range is similar to other fungi such as *Sclerotinia scleortiorum, Verticillium dahlia*, and *Rhizoctonia solani* but there is not a common understanding of the molecular basis of this host range or how this may impact the genome.

*B. cinerea* exists in diverse environmental conditions in an array of developmental forms such as mycelia, micro- and macro-conidia, chlamydospores, sclerotia, apothecia, and ascospores (Coley-Smith et al., 1980; Lorbeer, 1980). Sclerotia provide *B. cinerea* with the ability to survive within a soil reservoir for many years. The diverse array of sporulation forms enables numerous dispersal avenues. In addition to a wide range of life-styles, this pathogen is also striking in the lack of large-effect resistance loci found within tested plant hosts. This suite of characters generates a pathogen which causes endemic crop losses and has been highly recalcitrant to genetic methods of control and chemical control requires complex interchanging of fungicides to prevent buildup of resistant genotypes. While previous work has shown extensive genomic variation between two isolates, there is little understanding of the genomic frequency of variation within a collection of isolates across the species (Staats and van Kan, 2012). Thus, a genome wide survey of genetic diversity in this pathogen may help to better devise appropriate control methods by understanding its life style and host range.

Recent genetic studies have shown that *B. cinerea* contains considerable genetic and phenotypic diversity and populations rapidly reshuffle genetic material (Buttner et al., 1994; Giraud et al., 1997, 1999; Fournier et al., 2002, 2013; Munoz et al., 2002; Kliebenstein et al., 2005; Rowe and Kliebenstein, 2007, 2008; Fournier and Giraud, 2008; Rowe et al., 2010; Amselem et al., 2011; Estavillo et al., 2011; Aguileta et al., 2012; Schumacher et al., 2012). Some individual gene studies and marker based studies using microsatellites have identified variation that may contribute to the extensive host range (Giraud et al., 1997, 1999; Fournier et al., 2002; Munoz et al., 2002; Rowe and Kliebenstein, 2007; Fournier and Giraud, 2008). Other studies have however found no significant signature for host specificity using a genome wide survey with AFLPs (Ma and Michailides, 2005). Similar contradictions were present across the same studies about the role of biogeography in structuring the measured diversity. This analysis has been complicated by the use of dominant markers and microsatellites that have uncertainty in ascertaining identity by descent and suggests that genomic resequencing may be a better approach to survey the genetic diversity in this species (Simonsen et al., 1995; Kruglyak et al., 1998). Similarly both approaches only sample a small fraction of the genome and are unable to give a full genomic view of recombination or diversity. As such, by conducting a full genomic sequencing of a collection of isolates, we are working to provide a full genomic view of diversity and recombination within the species.

The presence of recombination in combination with a large pool of standing genetic variation may extend the genetic potential available to *B. cinerea* and contribute to the broad host range of *B. cinerea* the species. This recombination may arise from two potential sources within *B. cinerea*. First, shuffling may be via meiotic recombination which is supported by the identification that *B. cinerea* has a single mating type gene that has at least two alleles, and a sexual cycle requiring a partner of opposite mating type (Faretra et al., 1988; Faretra and Pollastro, 1996; Amselem et al., 2011). While the sexual cycle has been observed in the laboratory, no reported sexual morphs have been found within the field, potentially due to its small size leading to an ascertainment bias against its identification (Alfonso et al., 2000). Additionally, wild-collected isolates of *B. cinerea* have shown no heterozygosity at any measured SNPs suggesting that the field isolates are all haploid and that any diploid form must be rapidly resolved to the haploid if there is field meiosis (Rowe and Kliebenstein, 2007). Another possibility is that vegetative fusion may play a role in allowing genetic shuffling in the field via mitotic recombination (Glass et al., 2004; Beever and Weeds, 2007). Thus, while existing data shows that recombination contributes to the standing genetic diversity of *B. cinerea* it remains to be clarified if this recombination arises from sexual or parasexual recombination or to what extent recombination occurs. A genomic survey of breakpoint identification will provide insights into the level of recombination within *B. cinerea*. Further, an assessment of diversity at vegetative incompatibility loci may allow an insight into the potential role of parasexuality in this recombination.

To measure the genomic diversity found in the nuclear and mitochondrial genomes of *B. cinerea* and to begin mapping variation in recombination across the genome, we conducted a pilot study where we resequenced the genome of 13 isolates (Table S1). These 13 isolates were chosen to provide a representative sample of the breadth of diversity in a larger collection of isolates as assessed by the genetic variation at two cell-wall-degrading polygalacturonase loci, *BcPG1* and *BcPG2* (Rowe and Kliebenstein, 2007). This set of isolates provides us the ability to conduct a pilot study to estimate the level of recombination across the genome within the species and potentially to assess what the source of this recombination may be. In addition, we re-sequenced the genome of one of these isolates using four replicated cultures that had been separately maintained for a decade in two labs, to assess the potential rate of spontaneous mutation accumulation under standard lab practices. Our analysis of major effect polymorphisms within these isolates found 13 major effect polymorphism clusters with an enrichment in loci potentially controlling vegetative incompatibility. Sequencing these genomes focuses on the goal of determining the level of standing genetic variation in *B. cinerea* and how frequently it is shuffled by recombination to initially assess if this genomic architecture could in part contribute to the species broad host range.

## Materials and methods

### Sequencing

Isolates were grown on standard potato dextrose agar (PDA; Difco, Becton, Dickson and Company, East Rutherford, NY) with cellophane on the surface to prevent hyphal growth into the media. Information on isolate collection and sources is presented in Table S1. Young hyphae were collected from the leading edge of the mycelial mat using a sterilized spatula, and ground using stainless steel ball-bearings in P1 buffer from a Qiagen Plant DNeasy kit (Venlo, Netherlands). High quality DNA was obtained by following the manufacturer's recommendations for fungal DNA extraction. Sequencing libraries were constructed using a unique in-line 5′ bp barcode and pooled in groups of eight isolates. Pooled and multiplexed DNA libraries were sequenced on a total of four lanes using either an Illumina GAIIx or HiSeq machine. Post-sequencing, DNA libraries were de-multiplexed by their unique barcode for downstream analysis.

### Sequence clean-up, alignment, and variant calling

Raw sequence data was cleaned-up (quality threshold of 30) using Fastq-mcf from the ea-utils package (http://code.google.com/p/ea-utils), and 9 bp were trimmed from the start of each read. Sequences were aligned using STAMPY (Lunter and Goodson, 2011), with default parameters, to the T4 reference sequence (Staats and van Kan, 2012). Supercontigs were placed on 16 chromosomes for plotting purposes only by alignment to *Sclerotinia sclerotiorum* using PAGIT (Swain et al., 2012). Alignment and SNP calling of mitochondrial DNA was implemented as described for genomic DNA, except the reference sequence from BO5.10 was used (Staats and van Kan, 2012).

Samtools (Li et al., 2009) and Picard (http://sourceforge.net/projects/picard/) were used to prepare the alignments for determining polymorphisms using GATK's UnifiedGenotyper (Depristo et al., 2011). GATK and Picard software was used to remove duplicate sequence reads, recalibrate base quality scores, and locally realign regions around indels. All polymorphisms in both BO5.10 (82,212 bp) and the polygalacturonase genes (5492 bp × 13) were visually checked using SAVANT (Fiume et al., 2010).

Initially, the first round vcf file produced by GATK UnifiedGenotyper was filtered by removing anything with < 6X coverage and quality threshold of 30, which gave zero false positive SNPs when compared to known sequence of the *BcPG* genes (Rowe and Kliebenstein, 2007). However, there were ~25% false negative SNP calls for the highly diverse genes *BcPG1* and *BcPG*3. After additional filtering by mapping quality (MQ) of < 85, the majority of SNPs were called but three false positives were found. These were removed by a second Indel realignment. However, three false negatives were still found in *BcPG1*, in a difficult to call region of the Grape isolate, caused by clustering of four polymorphisms (three SNPs and an Indel) within a 16 base region. This was out of a total of 62 polymorphisms (GenBank: EF195807.1) and only when Indels were included. Similar polymorphisms were also not called in the isolates GLO1, Acacia and A517. Calling all polymorphisms as haploid (ploidy 1 setting in UnifiedGenotyper) removed these false negatives and all polymorphisms in the *BcPG* genes were called without any false positives.

The majority of initial heterozygous calls were removed during filtering. Those that remained, upon visual inspection, were determined as ‘hard to validate’ for example due to location of more than three SNPs and an indel within 10 bp region. As such, and due to the improved call rate, polymorphism calling by UnifiedGenotyper was carried out with the option for haploid data.

Variant annotation and effect prediction was performed using SnpEff version 3.4 (Cingolani et al., 2012) based on the T4 gene models for genomic and BO5.10 for mitochondria DNA (http://www.broadinstitute.org, *B. cinerea* BO5.10 mito; Staats and van Kan, 2012).

### Phylogeny

Consensus sequences were created and converted to fasta format from called SNPs using GATK before BIONeighbor-joining radial phylogenetic trees were determined based on pairwise SNP differences using SEAVIEW (Gouy et al., 2010).

### Spontaneous mutation

The four independently propagated Grape isolates were aligned to a consensus extracted via GATK from the Grape genomic and mitochondrial sequence. Although ~5000 variants were originally called, upon closer inspection these were revealed to be due to low coverage regions. When the coverage cut-off was reduced to three reads the remaining variants could be visually checked using SAVANT and were found to be caused by regions of no coverage where the program reverted to the assumed T4 sequence, as such no genuine variants were detected.

### Diversity and clustering analysis

Non-synonymous and synonymous polymorphisms were extracted from the diversity data generated by GATK UnifiedGenotyper as vcfs using SnpEff, followed by conversion to fasta consensus sequence for each contig using GATK. Non-synonymous and synonymous diversity was calculated as S (segregating sites) per contig using 10 kb sliding windows and 200 bp steps in DnaSP v5 (Librado and Rozas, 2009).

Major effect polymorphisms were extracted from the diversity data as vcfs using SnpEff and summed per gene. Sliding window plots were made of major effect polymorphisms per 10 gene window. A binary version of this data, where all polymorphisms per gene were altered to one polymorphism per gene was also created to visualize areas of interest more easily and to minimize the impact of individual loci with multiple major effect polymorphisms.

Proteins with non-synonymous SNPs and the translated T4 reference were blasted using Blast2GOPro from the Blast2go suite (Conesa et al., 2005) to obtain GO annotations. The extracted GO terms from the two datasets were analyzed using WEGO (Ye et al., 2006), which uses the Pearson Chi-sqaure test to show significant relations between two input datasets.

### Vegetative incompatibility assays

All isolates were grown from spores stored in 10% glycerol solution at –80°C. Stock solution was diluted in filter-sterilized ¼strength grape juice and plated on 39 g/L potato dextrose agar (PDA), and grown at 25°C, 12 h light/12 h dark. Plugs (~4 mm^2^) cut from the static region of the colonized PDA were transferred to new 15 cm PDA petri dishes. One plug was transferred per quadrant, for four paired isolate interactions per plate.

After 3–5 days of growth at 25°C, 12 h light/12 h dark, heterokaryon incompatibility was assessed as the formation of a melanic barrage (demarcation zone) at the mycelial interface of two genetically distinct isolates, due to accumulated dying cells. Interactions visible as growth arrest or faint, partial pigmentation were scored as a weak incompatibility phenotype. We examined 3–6 interactions per genotype pair, and reported the average observed phenotype.

### Recombination breakpoint analysis

RDP4 (Martin et al., 2010) was used to detect recombination in the 13 isolates plus the T4 reference. Default parameters were used for the seven programs RDP (Martin and Rybicki, 2000), GENECONV (Padidam et al., 1999), Bootscan (Martin et al., 2005), Chimera (Posada and Crandall, 2001), SiScan (Gibbs et al., 2000), MaxChi (Smith, 1992), and 3Seq (Gibbs et al., 2000). Recombination was considered to be real if detected by four or more of these programs using a Bonferroni corrected *P*-value cut off of 0.05.

## Results

### Genome sequencing and polymorphism control

To survey genomic diversity in *B. cinerea*, 11 isolates were sequenced with GAIIx 100-bp Illumina paired-end reads to obtain a mean 45-fold coverage (31–56X) and five isolates were sequenced by Illumina HiSeq 2000 100-bp paired-end reads to obtain a mean 90-fold coverage (75–133X). Information on isolate collection and sources is presented in Table S1. Reference based assemblies to call polymorphisms were by alignment to the previously sequenced *B. cinerea* isolate T4 for nuclear genomic and BO5.10 for mitochondrial genomic analyses (http://www.broadinstitute.org; *B. cinerea* T4; http://www.broadinstitute.org; *B. cinerea* B05.10; Staats and van Kan, 2012). This reference was selected, over the alternative previously sequenced reference BO5.10, as it had fewer and longer super-scaffolds (56 and N_50_ of 1.71 Mbp vs. 82 with N_50_ of 970 kbp; Staats and van Kan, 2012). Additional variant calling filters were applied to maximize the quality of the genome sequences generated. We optimized the filters to maximize polymorphism accuracy by empirical comparison to the previously sequenced cell-wall-degrading polygalacturonase genes, *BcPG1-3*, that had been sequenced in the same isolates (Rowe and Kliebenstein, 2007). We also utilized the empirical information available from alignment and polymorphism calling when comparing the reference sample BO5.10 to our BO5.10 sample. Additionally, all sequences were visually checked using Savant (Fiume et al., 2010). Using this approach our final settings were set to produce no false positives or false negatives amongst the *BcPG1-3* sequences. Analysis of the mitochondrial genome of our BO5.10 sequence against the published BO5.10 mitochondrial sequence found one false positive SNP and three false negatives using these settings across 153,000 bp (which were manually confirmed). All four of these polymorphisms were unique to the published BO5.10 genome sequence and not present in any other isolates suggesting that they are likely low coverage errors. Thus, when combining all of the empirically tested genes, our false positive rate has an upper bound of one per 153 kb while our false negative rates have a maximal bound of three per 153 kb. These error rates are acceptably low. All sequence polymorphism information is available in a vcf format at doi: 10.5061/dryad.4vd70.

### Clonal propagation and spontaneous mutation accumulation

Previous studies have suggested that the study of natural variation in virulence amongst pathogens may be influenced by the potential accumulation of spontaneous mutations during lab cultivation as clonal propagates have been noted to vary for virulence (Jeon et al., 2013). However, recent work in several species has shown that clonal propagates are not showing elevated mutagenesis (Kohn et al., 2008; Park et al., 2010). To test if there was any evidence for clonal propagation in *B. cinerea* that could be linked to mutation accumulation, we resequenced the genomes of four clones that had been experimentally separated from one isolate (Grape) and independently propagated for a decade, in two separate labs, on different continents (US and UK). The genome resequenced samples were then realigned both to the T4 reference and also to the sequence of the progenitor Grape isolate as described for the other isolates. All potential variation between the four independent Grape genomes was manually investigated and found to be solely present in areas lacking sufficient coverage. Thus, we could not identify any *de novo* polymorphisms between the genomes of these four independently propagated Grape isolate clones after controlling for lower coverage thresholds. As such, the Grape strain of *B. cinerea* shows no evidence for the accumulation of spontaneous mutations over at least a decade of separate propagation under lab conditions. This suggests that *B. cinerea* has a similar mutation rate as *Sclerotinia sclerotiorum* and *Magnaporthe oryzae* which indicates that mutation rate is not the main driver in the level of genomic diversity we identify in our isolates as compared to these other fungi (Kohn et al., 2008; Park et al., 2010).

### Elevated nuclear genomic diversity

**Figure 1:**
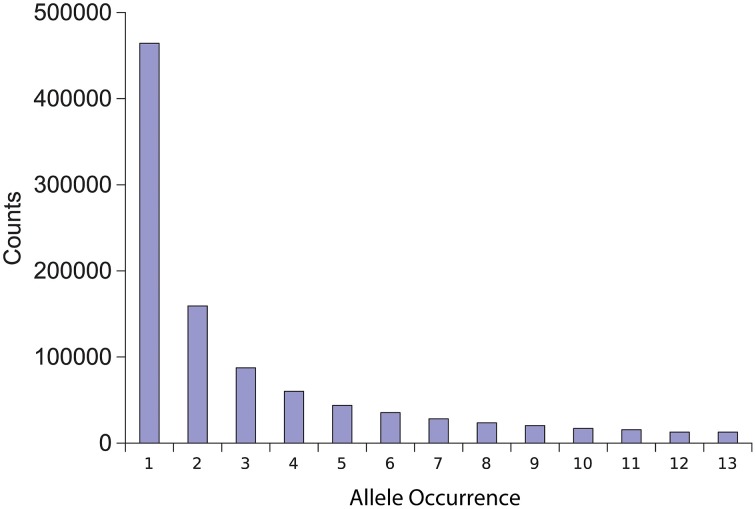
**Allele diversity in comparison to reference T4**. The number of isolates that share a polymorphic allele in comparison to the reference T4 isolate was plotted against the total number of polymorphisms with that level of diversity.

To identify genome-wide polymorphism levels in the nuclear genome, we aligned the genomes of the above isolates to the previously sequenced reference T4. This analysis identified 980,329 unique SNPs and 157,936 unique Indels across all isolates with between 144,109 and 403,608 polymorphisms per isolate (Table 1). There was no significant correlation between the level of sequence variation in an isolate and the depth of sequencing (Table 1, *P* = 0.09, *R* = 0.52, *N* = 12). Any appearance of a correlation was solely driven by the Grape isolate being both more sequenced and generally more diverged. Excluding this single outlier abolished any relationship between sequence variation and sequencing depth (Table 1, *P* = 0.99, *R* = 0.002, *N* =11). Aligning to the T4 reference also found extensive insertion and deletion polymorphisms. On average there were 12,560 deletions (7838–21,166) and 13,966 insertions (8492–25,137) per isolate in comparison to T4 (Table 1). Combining the polymorphisms (both SNP and indel) from all pairwise combinations of isolates yielded an average of at least one polymorphism between the isolates every 36 bp, giving an average density of 28 polymorphisms per kb within the population. Plotting the number of isolates that share a specific polymorphism in comparison to T4 showed that most alleles were of minor frequency with the major peak being a single isolate having each specific difference in comparison to T4 (Figure 1). These estimates for genomic diversity within *B. cinerea* are higher than that found so far in species with detailed whole genome resequencing projects such as *Arabidopsis* (Cao et al., 2011; Long et al., 2013), Humans (Altshuler et al., 2010), and Yeast (Schacherer et al., 2009).

**Table 1:** **Genomic polymorphism summary**.

**Polymorphism**	**Total**	**Isolate**												
		**Acacia**	**A517**	**BO5.10**	**1.01.12**	**Grape**	**GLO1**	**Geranium**	**Katie tomato**	**Davis navel**	**Rose**	**Noble rot**	**UKR**	**1.01.06**
SNPs	980,329	216,299	335,768	173,741	202,270	357,305	213,332	205,957	127,779	180,103	166,813	169,336	240,348	172,830
Indels	157,936	28,232	39,506	20,843	26,030	46,303	28,064	24,380	16,330	22,044	21,823	19,990	29,151	22,141
Insertions	87,489	15,039	20,751	10,820	13,724	25,137	14,990	12,568	8492	11,424	11,529	10,197	15,354	11,536
Deletions	70,447	13,193	18,755	10,023	12,306	21,166	13,074	11,812	7838	10,620	10,294	9793	13,797	10,605
Largest insertion	58	58	57	56	58	58	58	56	56	56	57	56	59	56
Largest deletion	74	74	74	72	73	74	73	72	72	73	72	73	72	73
Change every (bp)	36	170	110	213	182	103	172	180	288	205	220	219	154	213
Changes per kb	28	6	9	5	5	10	6	6	3	5	5	5	6	5
Missense/Silent	0.755	0.705	0.689	0.737	0.755	0.673	0.733	0.741	0.721	0.731	0.745	0.741	0.719	0.735
Missense	98,400	21,831	34,264	18,091	21,529	34,489	22,031	22,285	12,807	18,700	17,589	18,049	25,784	17,916
Nonsense	1255	180	264	126	222	290	157	188	85	155	130	143	216	150
Silent	130,295	30,950	49,709	24,562	28,535	51,215	30,066	30,096	17,766	25,577	23,596	24,355	35,839	24,393
Major effect changes	5963	1113	1412	717	1037	1613	982	877	494	772	848	683	1090	743
Sequence depth		92	55	46	85	141	78	52	59	52	78	33	13	56

The total number of genomic polymorphisms per polymorphism type detailed per individual genotype in comparison to the T4 reference genome. The total shows the unique occurrence summation across all genotypes. Largest insertion and deletion are in bp.

To investigate the potential functional consequence of these polymorphisms, we focused on polymorphisms within coding regions of predicted genes. This found 98,400 missense (42.8%), 1255 nonsense (0.5%), and 130,295 (56.7%) silent polymorphisms. The missense/silent ratio of 0.76 is in line with that found using the three published *B. cinerea* genomes (Amselem et al., 2011; Staats and van Kan, 2012; Blanco-Ulate et al., 2013). Using SnpEff (Cingolani et al., 2012) we extracted polymorphisms predicted to have major effects (protein truncation, loss of function or triggering nonsense mediated decay) on gene function using the annotation from T4 (http://www.broadinstitute.org; *B. cinerea* T4). This found 5963 polymorphisms likely to have a high impact on the function of 1801 genes (out of a possible 10,401 genes annotated in the T4 reference). Thus, 17% of the annotated genes in *B. cinerea* have at least one major effect polymorphism (polymorphisms that lead to a predicted functional knockout) in this limited sample of isolates and the genetic diversity is likely to have significant phenotypic impact on the processes mediated by these genes.

### Genome wide evidence of recombinational shuffling

**Figure 2:**
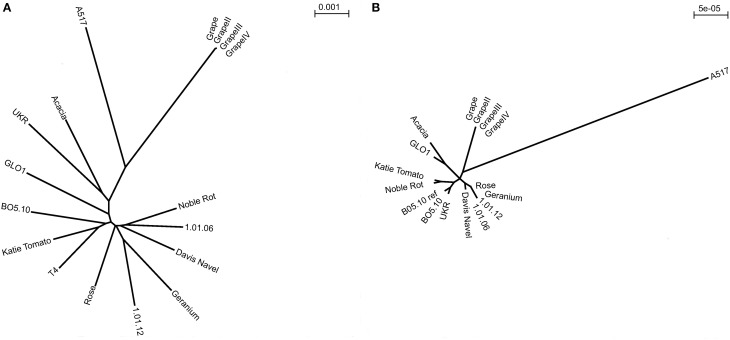
**Radial neighbor-joining phylogenetic trees of nuclear and mitochondrial diversity within *B. cinerea*. (A)** Unrooted genomic phylogeny determined based on pairwise SNP differences in the alignments of 17 *B. cinerea* strains to the reference T4 (including T4 reference and three replicate lineages independently propagated from the Grape isolate). Branch lengths are proportional to the number of segregating sites that differentiate each pair of isolates. **(B)** Unrooted mitochondrial phylogeny determined based on pairwise SNP differences to B05.10 in the alignments of 17 *B. cinerea* strains (including BO5.10 reference and three replicate lineages independently propagated from the Grape isolate). Branch lengths are based on very few polymorphisms and are proportional to the number of segregating sites that differentiate each pair of isolates.

To assess the genomic relationships amongst this collection of isolates, we used the genome wide diversity to plot the relationships of the isolates via a Neighbor-joining phylogenetic tree (Figure 2A). This was done using pairwise SNP differences to T4 to generate the alignments. The un-rooted radial phylogeny of the 17 isolates (including the reference T4) showed no statistically supported evidence of clustering indicating that there is no genetic isolation amongst the isolates. Of the isolates sampled, Katie Tomato showed the fewest and A517 the most SNP differences to the reference T4. The only genomes that were significantly associated were those from the repeated resequencing of independently propagated Grape isolate clones (Figure 2A).

We also used the genomic variation to directly search for recombination breakpoints using the package RDP4 (Martin et al., 2010). Analyzing the data on a per contig basis allowed us to identify 7735 potential recombination breakpoints using a Bonferroni corrected *P*-value cut off of 0.05 (Table S2). This shows that within this collection of isolates, there is evidence for a recombination breakpoint every 5.4 kb. There was significant variation in local recombination rates with a low of one breakpoint per 9.7 kb on contig 11.0 and a high of a breakpoint every 0.8 kb on contig 15.3 (Table S2). This shows that there is significant evidence for recombination amongst isolates within this species. This analysis suggests extensive recombination amongst these *B. cinerea* isolates on a genome wide level and is in direct contrast to other published plant pathogens that show extensive clonality in their genome or phenotypic variation (Blandon-Diaz et al., 2012; Wicker et al., 2013).

### Mitochondrial genome polymorphisms

Using the sequencing data, we next reconstructed the mitochondrial genomes in each of the isolates to compare the mitochondrial variation to the nuclear genome variation and obtained a mean 205-fold coverage (163–244X). Aligning the mitochondrial genome of the 13 isolates to the 82 kb of mitochondrial sequence from BO5.10 identified 53 SNPs, 52 insertions, and 32 deletions (Table 2). The BO5.10 mitochondrial genome was used because the T4 mitochondrial sequence was not published. This showed that mitochondrial genomic variation was approximately 20-fold lower than for the nuclear genome with an average polymorphism every 600 bp from the combined isolates. These SNPs included six missense and five silent polymorphisms within ORFs. Using SnpEff to identify major effect polymorphisms, nine mitochondrial polymorphisms were predicted to have large functional consequences in six genes (Table 3). These six genes include three genes within the NADH dehydrogenase complex, an ATP synthase subunit, LAGLIDADG endonuclease and NUMOD endonuclease. The LAGLIDADG and NUMOD endonuclease genes are self-mobile inteins/homing endonucleases that act like transposons suggesting that their major effect polymorphisms may affect the movement of these genes within the species (Stoddard, 2005). A larger collection of isolates will be required to test if there are different copy numbers and/or mobility of these mitochondrial transposons within the species. Thus, while the mitochondrial genome has less genetic diversity than the nuclear genome, there is still the potential for this genetic diversity to influence phenotypic diversity in the pathogen.

**Table 2:** **Mitochondrial polymorphism summary**.

**Polymorphism**	**Isolate**												
	**Acacia**	**A517**	**BO5.10**	**1.01.12**	**Grape**	**GLO1**	**Geranium**	**Katie Tomato**	**Davis Navel**	**Rose**	**Noble rot**	**UKR**	**1.01.06**
SNP	8	34	1	4	10	7	4	4	3	4	3	2	6
Insertion	15	25	2	5	7	9	8	9	10	8	10	15	13
Deletion	2	20	0	5	6	4	5	2	5	5	2	3	6
Rate (bp)	3288	1040	27,404	5872	3574	4110	4836	5480	4567	4836	5480	4110	3288

The total number of genomic polymorphisms per polymorphism type detailed per individual genotype in comparison to the mitochondrial sequence of BO5.10. Change rate is distance in bp between each polymorphism.

**Table 3:** **Mitochondrial genes with major effect polymorphisms**.

**Gene ID**	**Gene description**	**PFAM**
BC1G_20003	NADH-ubiquinone/plastoquinone oxidoreductase chain 4L	PF00420.19
BC1G_20006	NADH-ubiquinone/plastoquinone oxidoreductase	PF00507.14
BC1G_20007	NADH-Ubiquinone/plastoquinone (complex I)	PF00361.15
BC1G_20010	ATP synthase A chain	PF00119.15
BC1G_20011	LAGLIDADG endonuclease	PF00961.14
BC1G_20021	NUMOD1 domain	PF07453.8

List of six mitochondrial genes which have major effect polymorphisms within the sequenced isolates (frame shift, start lost, stop gained polymorphisms).

Within Fungi, mitochondria can either show uniparental inheritance or biparental inheritance with fusion and recombination (Basse, 2010). Phylogenetic analysis of the mitochondrial diversity suggested four clusters based on a total of 90 SNPs found amongst the isolates (Figure 2B). Although there was more apparent clustering in the mitochondrial phylogeny than found using the nuclear genome, it was not sufficient to support either of the potential patterns of mitochondrial inheritance. Interestingly, and without correlation to sequencing depth (Table 2), isolate A517 shows elevated mitochondrial variation in comparison to the other 12 isolates and accounts for 27% of the total mitochondrial variation, which is reflected in the phylogeny. This elevated mitochondrial diversity rate in A517 is significantly greater than the nuclear genome diversification of this isolate. It remains to be seen if there are any consequences of this elevated mitochondrial diversity. All of the independent propagates of Grape clustered given their lack of diversity. Thus, it is possible to identify significant levels of mitochondrial genomic diversity in *B. cinerea* and this may not reflect the diversity in the genome. Future studies are required to test if this mitochondrial diversity impacts the host/pathogen interaction.

### Genomic distribution of polymorphisms

**Figure 3:**
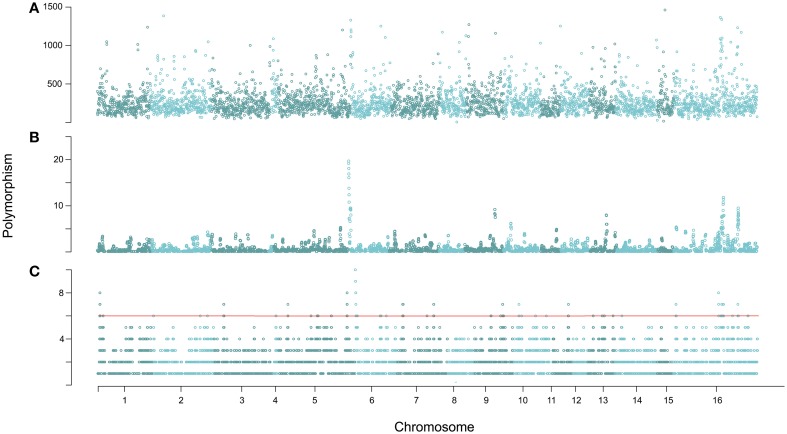
**Genomic distribution of polymorphism amongst the 14 isolates. (A)** A sliding window analysis of genomic polymorphisms per 10 kb across the genome is shown. **(B)** Shown is a 10 gene sliding window analysis of the number of genes with major effect polymorphisms based on comparison to the isolate T4. **(C)** Again a 10 gene sliding window analysis of the number of genes with major effect polymorphisms based on comparison to the isolate T4, but with polymorphisms per gene given a binary value. This enabled clusters of seven or more genes in 10 gene windows affected by major effect polymorphisms to be visually determined more easily. Thousands of permutations of the major effect polymorphisms established a significance threshold of six genes per window, which is drawn for reference. No one isolate is key cause of these clusters.

Given the high level of genomic diversity in *B. cinerea*, we sought to determine if this diversity was randomly distributed across the genome or showed evidence of clustering. The chromosomes of *B. cinerea* have not been delineated, as such the program PAGIT (Swain et al., 2012) was used to superficially overlay all contigs on chromosomes by comparison to *Sclerotinia sclerotiorum* (http://www.broadinstitute.org/sclerotinia_sclerotiorum/maps). This fungus is highly co-linear to *B. cinerea* with an identical chromosome number (Shirane et al., 1989; Amselem et al., 2011), and has an almost complete chromosome map. This created a visualization framework by placing the contigs on 15 chromosomes, with all unplaceable contigs being assigned into artificial chromosome 16 (as they were in the *S. sclerotiorum* used). The distribution of polymorphisms amongst contigs, placed on these superficial chromosomes, displayed regions or peaks of high density polymorphism, across all chromosomes (Figure 3A and Table S2). Change rates varied greatly per isolate and per contig, with the highest being one polymorphism every 40 bp in contig 15.3 of Grape and the lowest one change every 27,839 bp in contig 4.1 of Katie Tomato (Table S2).

To further dissect differences in the distribution of polymorphisms likely to affect function, the locations of major effect (frame shift, start lost, stop gained polymorphisms) were plotted within a 10-gene sliding window across the genome (Figure 3B). Plotting the major effect diversity as binary values rather than total number of major effect polymorphisms highlighted clearer clusters for gene dissection. This identified 13 windows where there were more major effect polymorphisms clustered than would be expected by random chance given this collection of isolates (Figure 3C, significance tested by a permutation test). Each of these clusters contains seven or more genes with high impact polymorphisms within a 10-gene window (Figure 3C).

An analysis of these polymorphisms showed that they were mostly moderate frequency polymorphisms (i.e., each allele occurring in more than one isolate) and not singleton polymorphisms where the alternate allele exists in only one particular isolate (Table S3). Gene families (determined from the T4 annotation) over-represented in these 13 major effect polymorphism clusters, showed statistically significant enrichments for heterokaryon incompatibility genes (5 of 67; *P* < 0.001 via χ^2^) and Nacht domain protein encoding genes (4 of 32; *P* < 0.001 via χ^2^) which have previously been associated with vegetative incompatibility in fungi (Koonin and Aravind, 2000; Paoletti et al., 2007; Zhao et al., 2015). Together, these five heterokaryon incompatibility genes and four Nacht domain protein encoding genes were present in six of the 13 major effect clusters. To test if the occurrence of these genes in the clusters was due to the particular gene families having higher diversity rates than the average gene, the rate of non-synonymous polymorphisms in all the vegetative incompatibility and Nacht domain genes was compared against the genome average. Neither gene family showed an elevated level of non-synonymous polymorphisms in comparison to the genomic average (8 of 32 NACHT genes and 11 of 67 HET proteins contained non-synonymous SNPs). Thus, the enriched presence of these genes within these major effect clusters is not being driven by elevated diversity of the gene family in relation to the rest of the genome (Table S4).

Five of the remaining seven clusters (that did not include heterokaryon incompatibility genes and or Nacht domain protein encoding genes) all contained one or more transporters of the Major Facilitator Superfamily (8 out of 287). Although this does not reflect enrichment of this family in the clusters, it suggests that transporters may be another target of genetic diversity. These transporters may play a role in controlling the pathogen's resistance to plant defense compounds (Stefanato et al., 2009). Alternatively, they may be required to allow the export of fungal phytotoxins that target the plant to enhance virulence (Stergiopoulos et al., 2002; Choquer et al., 2007). Future work with larger populations will be required to test the potential function of these transporters in any plant/pathogen interaction.

### Biological conformation of heterokaryon incompatible loci phenotype for loci within major effect polymorphism clusters

**Figure 4:**
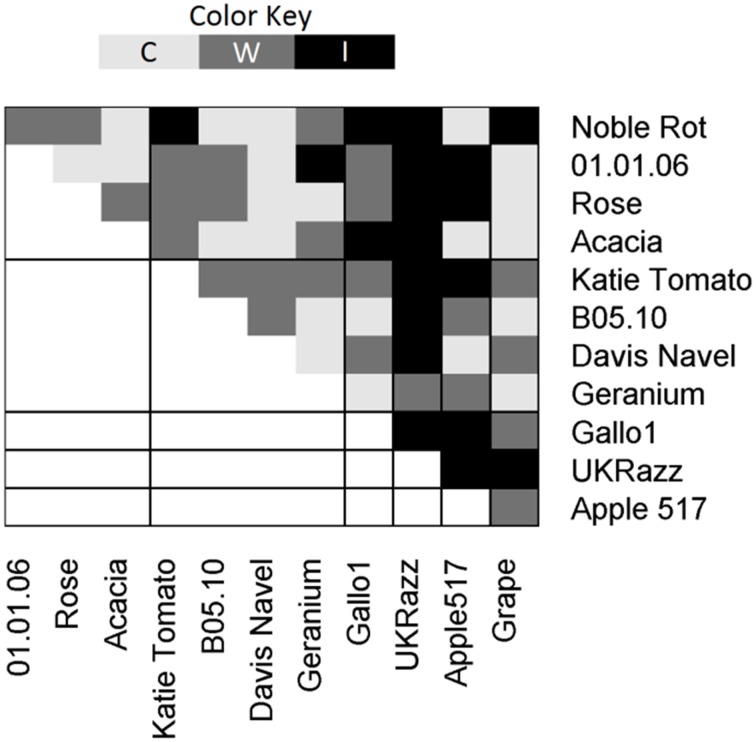
**Pairwise Vegetative Incompatibility**. All pairwise crosses were tested in at least triplicate and the incompatibility was scored as compatible (C), weakly incompatible (W), and incompatible (I) and results are shown above. χ^2^ was used to test if there was a difference in incompatibility when crossing within or between the Major Effect Haplotypes (χ^2^ = 4.9, *P* = 0.02, *N* = 66, *df* = 2).

To test if the major effect allelic diversity of the heterokaryon incompatible loci located within the major effect clusters could be controlling variation in vegetative incompatibility; the isolates were grouped based on their haplotypes at these loci (Figure 4 and replication information is provided in Table S6). We then conducted vegetative incompatibility assays between all pairs of isolates to test if the heterokaryon haplotype modulated vegetative fusion. In these tests, the incompatibility was significantly stronger when vegetative fusion was tested between isolates with different haplotypes than when they shared a haplotype (χ^2^ = 4.9, *P* = 0.02, *N* = 66, *df* = 2). All strong incompatibilities were found when vegetative fusion was tested between isolates that represent different haplotypes at the major effect clusters (Figure 4). In contrast, when vegetative fusion was tested within a haplotype group, there were no strong incompatibilities (Figure 4). This suggests that while we have likely identified some components controlling diversity in the vegetative fusion process, there are other vegetative incompatibility loci remaining to be identified potentially via sequencing a larger collection of isolates.

### Genomic distribution of non-synonymous and synonymous polymorphisms

**Figure 5:**
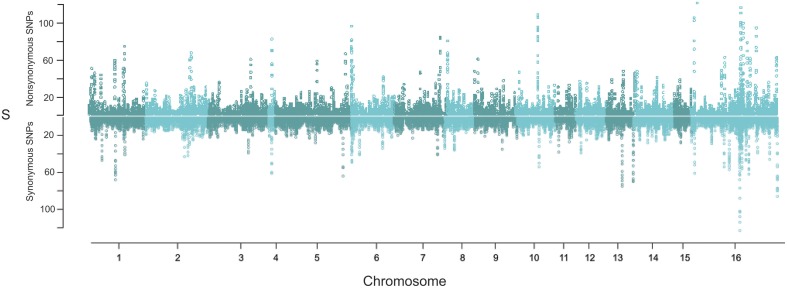
**Genomic distribution of non-synonymous and synonymous SNPs**. Polymorphisms were assigned as non-synonymous or synonymous amongst the 14 isolates (including T4) and then measured in a 10 gene sliding window as the number of segregating sites (S). The top mirrored plot shows the sliding window of non-synonymous polymorphisms while the bottom shows the corresponding synonymous analysis.

**Figure 6:**
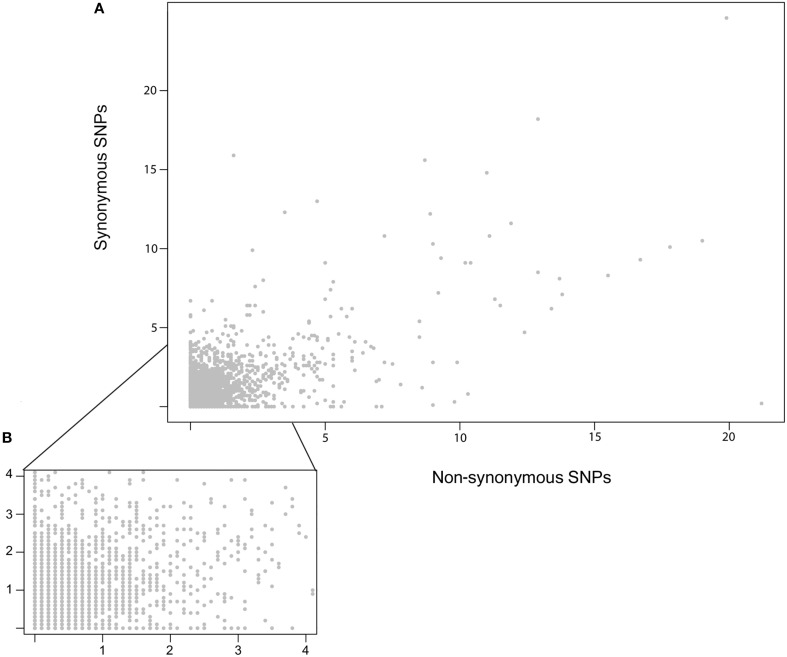
**Relationship of Synonymous vs. Non-synonymous SNP frequencies. (A)** An X/Y scatter plot showing the frequency of Synonymous vs. Non-synonymous SNPs per 10 kb bins. The x and y axis show the fraction of SNPs per kb for each window. No significant correlation was found using either pearson or spearman rank. **(B)** A zoomed in view of **(A)**, focused on the regions up to four polymorphisms per kb per 10 kb bin.

To compare the distribution of potentially neutral and non-neutral polymorphisms the genomic diversity in non-synonymous and synonymous polymorphisms was plotted as the number of segregating sites (S) within 10 kb sliding windows (Figure 5). This identified a number of regions with an elevated rate of clustering of non-synonymous SNPs. Notably, non-synonymous SNPs are abundant in the regions of major effect polymorphism (Figures 3, 5). Highly polymorphic regions of non-synonymous and synonymous SNPs appeared to occur in the same 10 kb bins as each other when visualized in a mirrored plot (Figure 5). However, in the majority of these regions of clustered polymorphisms, the rate of non-synonymous SNPs is noticeably higher (Figures 5, 6A). In contrast, there were only three regions of clustered polymorphisms where the synonymous polymorphism level was higher than the non-synonymous rate (one on chromosome 1 and two on chromosome 13). In contrast to the regions of elevated diversity, synonymous SNPs were more abundant overall across the genome in the regions of moderate or lower polymorphism rates (Figure 6B). This suggests that these regions of high non-synonymous polymorphism and major effect polymorphisms are non-random. Fully testing this hypothesis and assessing the potential for diversifying selection will require the sequencing of more isolates to generate a larger sample size.

To investigate non-synonymous SNPs further, as they are also likely to cause mutations which affect phenotype, we used Blast2GOPro from the Blast2go suite to test if there was enrichment for a particular function in genes with non-synonymous polymorphisms (Conesa et al., 2005). GO terms were extracted for both the translated T4 reference sequence and loci classified by presence or absence of non-synonymous SNPs. These two sets of GO terms were analyzed using WEGO (Ye et al., 2006), which uses the Pearson Chi-square test to show significant relations between two input datasets. GO enrichment analysis found that the proteins containing non-synonymous SNPs were involved in 46 of the ontologies that could be analyzed (Table S5 and Figure S1). Of interest amongst these (Table S5, highlighted in blue) were numerous non-synonymous SNP enrichments that were found in proteins associated with botcinin production. Botcinins are chemical toxins produced by *B. cinerea* that are virulence determinants (Dalmais et al., 2011) and as such are of interest in potentially determining isolate and crop specific interactions.

## Discussion

While previous work had shown significant genomic variation between two isolates, this had not assessed the potential frequency or level of diversity in the species (Staats and van Kan, 2012). Our genomic resequencing of 13 *B. cinerea* isolates revealed that the previous two isolates had only sampled a small range of the diversity found in this collection. Thus, we are still sampling new diversity at a high level (Amselem et al., 2011; Staats and van Kan, 2012; Blanco-Ulate et al., 2013). Importantly, there is a significant fraction of the polymorphisms that have moderate allele frequencies indicating that there is a large pool of standing variation. With the presence of extensive recombination in this species, this allows for this variation to be readily shuffled. Thus, we have only begun to sample the potential genetic diversity in this pathogen.

Even with this under-sampling, the level of diversity is higher than in any previously studied plant pathogen. For example, the sequencing of two *Blumeria graminis ff.* ssp. *hordei* isolates and comparison to the previously sequenced reference DH14 gave an approximate SNP change rate of one SNP per kb vs. 3–9 polymorphic SNPs per kb between individual isolates found within our study (Hacquard et al., 2013). Resequencing of *Blumeria graminis ff. ssp tritici* provided numbers of between 1.4 and 2.0 SNP per kb between genotypes (Wicker et al., 2013). This puts the estimates of genomic diversity in both plant pathogens lower than in *B. cinerea. One* potential reason for this level of genomic diversity within *B. cinerea* is that it may facilitate the broad host-range of the pathogen, including nearly every plant tested to date. Broader sampling of both *B. cinerea* as well as other broad and narrow host range plant pathogens is required to investigate if the level of genomic diversity may contribute to host-range diversity.

### Mitochondrial genomic variation

In animals, mitochondrial DNA has been shown to accumulate polymorphisms more rapidly than genomic DNA (Birky and Walsh, 1992; Birky, 1995). In our *B. cinerea* analysis, this relationship was reversed, with the genomic DNA having a nearly 20-fold higher diversity rate than the mitochondria (Figure 2). A low level of mitochondrial diversity has also been found in other fungi (Zhan et al., 2003). For example, a study of four strains of *Rhizophagus irregularis* found zero SNPs (Formey et al., 2012) and a study of *M. graminicola* found a similar diversity rate as we detected in *B. cinerea* mitochondria (Torriani et al., 2008). Unfortunately, measures of nuclear genomic diversity were not available in these studies so it remains to be seen if most fungi have lower mitochondrial diversity than nuclear genome diversity. Even with this low level of diversity, there were major effect polymorphisms in specific genes within the mitochondria suggesting diversity could cause phenotypic consequences.

### Observed field adaptation of *B. cinerea* is likely via selection of standing population diversity rather than elevated mutagenesis

Frequent studies focused on the agronomic control of *B. cinerea* infections in the field or within lab studies have suggested that the ability of *B. cinerea* to show resistance to fungicides or isolates to display variation might be caused by the accumulation of spontaneous mutations (Grindle, 1979; Kerssies et al., 1997; Yourman and Jeffers, 1999; Yourman et al., 2000, 2001). This mutation accumulation hypothesis has often been postulated to occur in fungi, viruses and prokaryotes (Drake et al., 1998; Lamb et al., 2008). However, recent work in in *Sclerotinia sclerotiorum* and *Magnoparthe oryzae* has shown that there does not appear to be an effect of lab culturing on mutation rate (Kohn et al., 2008; Park et al., 2010). Our genomic resequencing of four individual propagates detected no spontaneous polymorphisms in either the genomic or mitochondrial alignments. Each line was propagated on average at least four times per year but given the continuous formation of conidia in *B. cinerea* it is not possible to generate an accurate estimate of generation number. As such, it would appear that *Sclerotinia sclerotiorum, Magnoparthe oryzae* and *B. cinerea* do not have an elevated spontaneous mutation rate (Kohn et al., 2008; Park et al., 2010). This suggests that there is a large level of standing diversity within *B. cinerea* populations and that the observed field “evolution” against a fungicide or other agronomic control is likely a result of selection on massive standing diversity rather than *de novo* evolution.

### Vegetative incompatibility genes were found in the major effect polymorphism clusters

Our analysis of major effect polymorphisms within these isolates found 13 major effect polymorphism clusters with an enrichment in loci potentially controlling vegetative incompatibility. Filamentous ascomycete fungi can fuse their hyphae, fuse their nuclei, recombine and resolve back into haploid isolates in a process called parasexuality. This process is prevented by incompatibility loci called heterokaryon incompatibility loci which stimulate programmed cell death when incompatible hyphae fuse (Glass et al., 2004). These het (heterokaryon) loci have previously been shown to be under selection for diversity in other fungal species (Hall et al., 2010; Zhao et al., 2015). Further, they can prevent transmission of mycoviruses by hyphal fusion. Loss-of-function mutations at het genes in *N. crassa* do not affect the vegetative growth phenotype of mutants but these mutants fail to distinguish self from non-self and will form vigorous heterokaryons with strains with which they were formerly incompatible (Shiu and Glass, 1999). Noticeably, all genes containing HET-E-1 domains that are present within major effect polymorphism clusters contained major effect polymorphisms, i.e., variants that should abolish their function. However, these polymorphisms at independent loci were shuffled amongst the isolates with different genes being polymorphic in different isolates.

Biological confirmation of the major effect polymorphism within these heterokaryon incompatible loci showed that we could predict the vegetative fusion interaction using the haplotype at these het loci. This observation is in agreement with previous reports that suggested a potential role for parasexuality in *B. cinerea* diversity (Beever and Weeds, 2007). What this means in regards to reproductive barriers between isolates and how this may affect the adaptation to new plant hosts should become clearer as more isolates are resequenced.

## Future directions

Our genomic resequencing has shown that the broad-host range plant pathogen *B. cinerea* contains a high level of genomic diversity. Further, there appears to be shuffling of this polymorphism within the nuclear genome as evidenced by the lack of clonality. That *B. cinerea* has a large pool of shuffling standing genetic diversity may enhance its ability to adapt to new hosts. This high level of standing variation suggests that there is significant utility in the creation of a large *B. cinerea* mapping population. Future efforts will focus on conducting genome-wide association mapping within the pathogen to determine how this genomic diversity leads to phenotypic variation in the host-pathogen interaction. As such it will be interesting to assess how larger diversity studies in *B. cinerea* identify the link between genotype and phenotype in altering the plant/pathogen interaction.

### Conflict of interest statement

The authors declare that the research was conducted in the absence of any commercial or financial relationships that could be construed as a potential conflict of interest.
