# Supplementary material for: Whole genome resequencing of Botrytis cinerea isolates identifies high levels of standing diversity
Source: Front Microbiol. 2015 Sep 24;6:996. doi: 10.3389/fmicb.2015.00996 (PMC4585241; doi:10.3389/fmicb.2015.00996)
Supplement: Supplementary file 1 [file Table1.DOCX]

**Table S1. Isolate description and origin.**
