# Supplementary material for: Whole genome resequencing of Botrytis cinerea isolates identifies high levels of standing diversity
Source: Front Microbiol. 2015 Sep 24;6:996. doi: 10.3389/fmicb.2015.00996 (PMC4585241; doi:10.3389/fmicb.2015.00996)
Supplement: Supplementary file 2 [file Table2.DOCX]

**Table S2. Breakpoints and genomic change rate per contig.**

Distribution of breakpoints and changes per contig as determined from alignment to the T4 genomic sequence. Both the total polymorphism across all isolates and the individual isolate sample change rate (distance in bp between each polymorphism) are presented per contig.
