# Supplementary material for: Whole genome resequencing of Botrytis cinerea isolates identifies high levels of standing diversity
Source: Front Microbiol. 2015 Sep 24;6:996. doi: 10.3389/fmicb.2015.00996 (PMC4585241; doi:10.3389/fmicb.2015.00996)
Supplement: Supplementary file 3 [file Table3.DOCX]

**Table S3. Allele distribution of major effect polymorphisms for genes within the identified major effect polymorphism clusters.**

A 1 in the allele columns shows the presence of a major effect polymorphism (frame shift, start lost, stop gained polymorphisms) in that isolate with regards to the T4 genome. A 0 shows that the isolate contains the T4 functional allele. Genes predicted to be associated with vegetative incompatability are highlighted in yellow.
