# Supplementary material for: Whole genome resequencing of Botrytis cinerea isolates identifies high levels of standing diversity
Source: Front Microbiol. 2015 Sep 24;6:996. doi: 10.3389/fmicb.2015.00996 (PMC4585241; doi:10.3389/fmicb.2015.00996)
Supplement: Supplementary file 4 [file Table4.DOCX]

**Table S4. Vegetative incompatibility (Nacht and Het domain) enrichment within major effect clusters.**

Chi squared test for enrichment for Nacht and Het domains found within the major effect polymorphism clusters. The list of total genes for each category in the genome, the ones with a major effect polymorphism (NS/Major) and those found within a major effect polymorphism cluster are listed. Exp shows the number of genes expected with a random model.
