# Supplementary material for: Whole genome resequencing of Botrytis cinerea isolates identifies high levels of standing diversity
Source: Front Microbiol. 2015 Sep 24;6:996. doi: 10.3389/fmicb.2015.00996 (PMC4585241; doi:10.3389/fmicb.2015.00996)
Supplement: Supplementary file 5 [file Table5.DOCX]

**Table S5. WEGO analysis of nonsynonymous polymorphisms.**

Significant results from the WEGO analysis obtained from the Pearson Chi-sqaure test showing enrichment of GO annotations. For both datasets, the gene number, percentages and P-value of Pearson Chi-square test of each GO term are listed from left to right, followed by corresponding gene function, where available. Of particular interest were the Botcinins, which are highlighted in blue.
